# Phase II study of compensator-based non-coplanar intensity-modulated radiotherapy for Stage I non–small-cell lung cancer

**DOI:** 10.1093/jrr/rrz009

**Published:** 2019-04-11

**Authors:** Tomohiro Itonaga, Ryuji Mikami, Hidetsugu Nakayama, Tatsuhiko Saito, Sachika Shiraishi, Mitsuru Okubo, Shinji Sugahara, Norihiko Ikeda, Koichi Tokuuye

**Affiliations:** 1 Department of Radiology, Tokyo Medical University Hospital, 6-7-1 Nishi-shinjyuku,Shinjyuku, Tokyo, Japan; 2 Department of Radiation Oncology, National Center for Global Health and Medicine, 1-21-1 Toyama, Shinjyuku, Tokyo, Japan; 3 Department of Thoracic Surgery, Tokyo Medical University Hospital, 6-7-1 Nishi-shinjyuku,Shinjyuku, Tokyo, Japan

**Keywords:** IMRT, Stage I, NSCLC, compensator, radiotherapy, Phase II study

## Abstract

We conducted a Phase II study to evaluate the usefulness of compensator-based non-coplanar intensity-modulated radiotherapy (ncIMRT) for patients with surgically inaccessible Stage I non–small-cell lung cancer (NSCLC). Patients with pathologically proven or clinically diagnosed surgically inaccessible Stage I NSCLC were enrolled in this study from May 2011 to April 2014. These patients underwent ncIMRT of 75 Gy in 30 fractions regardless of the tumor location. The primary end point was 3-year overall survival, and the secondary end points were local control rate and treatment-related toxicities. A total of 48 patients (50 tumors) were enrolled in this study. Of the 50 tumors, the Stage T1 to T2 ratio was 31 to 19, and the ratio of tumors located in the central to peripheral areas was 11 to 39. During the median follow-up time of 35.9 months, the 3-year actuarial local progression-free and overall survival rates were 82.6% and 87.1%, respectively. No patients experienced toxicities of Grade 3 or greater. Standard-fractionated ncIMRT was effective and safe for patients with surgically inaccessible stage I NSCLC, regardless of the tumor location.

## INTRODUCTION

The natural course of lung cancer is unfavorable, with a 6% 5-year overall survival (OS) rate even for Stage I (Stage IA: T1N0M0 or Stage IB: T2aN0M0) non–small-cell lung cancer (NSCLC) [1]. The Japanese Lung Cancer Registry Study showed that the 5-year OS rates of 6295 patients with clinical Stage IA and 2339 patients with Stage IB were 82% and 67%, respectively, when surgical resection was performed [2]. Owing to the aging of the population, underlying lung diseases (e.g. severe emphysema), as well as other diseases (e.g. diabetes mellitus, heart diseases and cerebral vascular diseases), are becoming more common. The number of patients who refuse surgery is also increasing, which makes surgical treatment difficult for lung cancer patients. Furthermore, aging of the population has resulted in an increase in the number of patients who refuse surgery.

Stereotactic body radiotherapy (SBRT) has demonstrated excellent outcomes for 57 patients with Stage I NSCLC, which appears comparable to the outcome of surgery [3]. Although surgical resection is the most reliable treatment for Stage I NSCLC, SBRT is becoming an option for patients with surgically inaccessible tumors [4–6]. In this study, we used compensator-based non-coplanar intensity-modulated radiotherapy (ncIMRT), which is an IMRT optimized by using adequate non-coplanar beams. We therefore started a Phase II study of compensator-based ncIMRT for patients with Stage I NSCLC to evaluate its effectiveness and treatment-related toxicities.

## PATIENTS AND METHODS

### Patient eligibility

The Ethics Committee of Tokyo Medical University Hospital approved this study (Approval No. IRB-1625), and all patients gave written informed consent to participate in the study. The eligibility criteria included the following conditions: (i) age of 20 years or older; (ii) performance status of 0 or 1 according to World Health Organization guidelines; (iii) a diagnosis with NSCLC by cytology or histology, or a clinical diagnosis with NSCLC by findings on positron emission tomography (PET-CT), or a tumor that had increased by >25% by CT when a histological diagnosis was not made; (iv) clinical stage of T1–2aN0M0 according to the 7th UIBC TNM classification by CT or PET-CT taken within the past 40 days; (v) medically inoperable conditions determined by the cancer board (which consists of thoracic surgeons, medical oncologists, radiation oncologists and diagnostic radiologists). Patients with suspected mediastinal lymph node metastasis by PET-CT underwent biopsies of endobronchial ultrasound and had no metastasis confirmed pathologically. Patients who had previously received radiotherapy for the area near the tumor, had pleural effusion, or had a prognosis of <6 months, were excluded from the study.

### Radiotherapy methods

Treatment procedures have been described elsewhere and briefly described thereafter [7]. All patients were immobilized in the supine position using a body-fixed shell system (Pelvicast; Orfit Industries n.v., Wijnegem, Belgium) so as to be in exactly the same position and to reduce the tumor motion. Tumor motion was restricted to within 1.0 cm, by fixing the body shell system, allowing ncIMRT planning, otherwise the therapy would have been considered unsuitable. The planning CT was taken in both inspiration and expiration phases under normal breathing conditions. These images were transferred to the target drawing machine (MIM maestro ver. 6.1, MIM Software Inc., Cleveland, USA), and the clinical target volume (CTV) was delineated to cover the gross tumor volume with a 0.5 cm margin in all directions on the CT images taken during the expiration phase. The internal target volume (ITV) was then generated by piling CTV images taken in the inspiration phase. The planning target volume (PTV) was created by adding 0.5 cm in all directions to the ITV. Organs at risk (OARs), such as the left lung, the right lung, the heart, the spinal cord and the esophagus, were also delineated. The lung volume was defined as the entire lung parenchyma excluding the gross tumor volume (GTV). These images were sent to the treatment planning system (Xio ver. 4.6 system, Elekta AB, Stockholm, Sweden). In order to maintain daily positioning accuracy, we performed CT to adjust the isocentric position before every treatment during the first week. If the positioning errors were within 3 mm in all directions, the positioning check was performed once a week thereafter.

The prescribed dose was determined to be 75 Gy in 30 fractions over 6 weeks; 95% of the prescribed dose was set to cover 95% of the PTV. The prescribed doses were usually input as follows: minimum and maximum doses were determined to be 75 Gy and 85 Gy for both CTV and PTV; the ipsilateral lung volume exceeding 20 Gy and 10 Gy were determined to be <20% and <40%, respectively; The contralateral lung volume exceeding 20 Gy and 10 Gy were <5% and <10%, respectively. The maximal doses to the spinal cord and the esophagus were determined to be <40 Gy and <50 Gy, respectively. Five non-coplanar beams were usually arranged to reduce the lung doses and to avoid exceeding the dose limits to the OARs, such as the spinal cord and the esophagus, according to the previous report [7]. The dose to the bronchus was obtained by calculation using the dose distributions and was confirmed to be <3 ml at >75 Gy in 25 fractions [8]. Usually, three coplanar beams from below and two non-coplanar beams from above, or four coplanar beams from below and one non-coplanar beam from above were used. If these dose distributions were considered inadequate, 7 or 9 ports were selected. Treatment planning for the ncIMRT was calculated, using the treatment-planning machine, to fulfill the above conditions, and compensated filter data were generated. After the dose distributions that were calculated using the compensated filter data were approved by the radiation oncologist, the compensated filter data were sent to a company (.decimal, Inc, Sanford, FL, USA). The filters, which were made by the company according to the data, were tested for clinical use using the 3D radiation detector (Delta 4, ScandiDos AB, Uppsala, Sweden). Chemotherapy was not performed during radiotherapy.

### Evaluation and analysis

Patients had a follow-up appointment every 3 months after completion of radiation treatment. They underwent chest CT scans every 3 months for 2 years, and then every 6 months thereafter. When recurrence was suspected, tight follow-up CT or PET-CT was performed. The final judgment regarding recurrence was made by the cancer board. To evaluate the treatment method, the percentage of the total lung volume exceeding 20 Gy (V_20_) from the total lung dose–volume histogram (DVH) was calculated [9]. Tumors were divided into two groups: namely, central tumors and peripheral tumors, in accordance with the criteria of Timmerman [10]. Acute and late treatment-related toxicities were assessed according to Common Terminology Criteria for Adverse Events (CTCAE), ver.4.0.

In the beginning of this study, the primary end point was determined to be OS, and the secondary end points were local control and treatment-related toxicities. The null hypothesis of OS for the usual type of fractionated radiotherapy was chosen to be 60%, and of OS for ncIMRT was estimated to be 80%. Then, the required number of patients was calculated to be 41 patients when alpha and beta errors were 5% and 15%, respectively, using the SWOG statistical tool ‘One Arm Nonparametric Survival’ (SWOG Statistical Center, Seattle, WA, USA) based on Brookmeyer–Crowley method. All data were calculated from the day of completion of ncIMRT. Median values were compared by the Wilcoxon–Mann–Whitney test. Local control and OS rates were calculated using the Kaplan–Meier method, and differences were evaluated by the Log-rank test. Multivariate regression analyses using the Cox proportional hazards model were performed to find risk factors. A *P*-value of 0.05 or less was considered to indicate a statistically significant difference between groups. Data were analyzed using statistics software (R software v. 3.1.0, R Foundation for Statistical Computing, Vienna, Austria).

## RESULTS

Between March 2012 and March 2014, 48 patients with NSCLC (50 tumors) were enrolled and treated by ncIMRT at Tokyo Medical University Hospital. Patient characteristics are summarized in Table 1. Of the 50 tumors, 17 (34%) were diagnosed as NSCLC by pathological examinations, and the others by clinical data; 11 tumors (22%) were classified as central tumors and the remainder as peripheral tumors.

**Table 1.** Patient and tumor characteristics

Characteristic	No. of patients
**Sex**	
**Men**	**32**^a^
**Women**	**18**
**Age at diagnosis (years)**	
**Mean**	**79.5**
**Range**	**49–90**
**History of pneumonectomy**	
**Yes**	**22**
**No**	**28**^a^
**Performance status**	
**PS0**	**35**^a^
**PS1**	**15**^a^
**Histology**	
**Adenocarcinoma**	**11**
**Squamous cell carcinoma**	**4**
**NSCLC**	**2**
**Unproven**	**33**
**T-stage**	
**T1**	**31**
**T2**	**19**
**Tumor location**	
**Central**	**11**
**Peripheral**	**39**

^a^Includes patients who received two rounds of radiotherapy.

**Figure 1.**
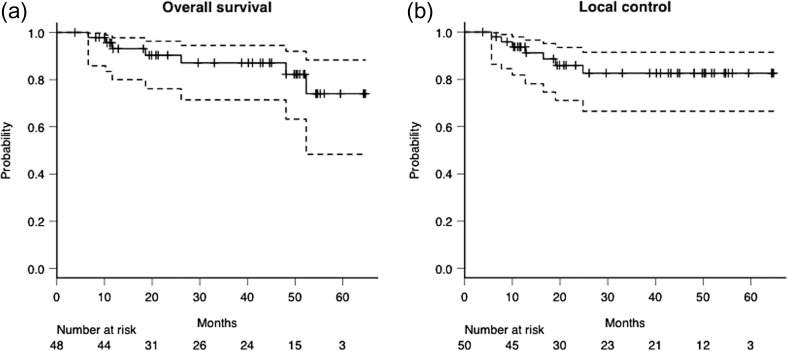
The overall survival of patients (**a**) and the local control of tumors (**b**) from the completion of cIM-SBRT, which were estimated using the Kaplan–Meier method, are shown. The 3-year local control and overall survival rates were 82.6% (95% CI, 66.5–91.4%) and 87.1% (95% CI, 71.4–94.5%), respectively.

The median follow-up period was 35.9 months (range, 3.8–64.7 months). The 3-year overall survival (OS) and local control (LC) rates were 87.1% (95% CI, 71.4–94.5%) and 82.6% (95% CI, 66.5–91.4%), respectively (Fig. 1). Seven patients (14.6%) demonstrated local failure during the median follow-up period of 12.7 months. Of these patients, two received re-irradiation and are surviving at the time of writing. Four patients requested palliative care, and the other requested treatment at other hospitals. Seven patients (14.6%) died after a median time of 18.6 months after the treatment. Five of these patients died of the progression of lung cancer, one of myocardial infarction, and the remaining one of aspiration pneumonia.

There was no significant difference in OS between central and peripheral tumors, T stages of T1 and T2, pathological proven and unproven tumors, sex, or age of <80 years versus >80 years by either univariate or multivariate analysis. Regarding LC, having a central tumor [hazard ratio (HR) 5.8, 95% CI: 1.2–27.6, *P* = 0.03] was the only significant risk factor among these factors on multivariate analysis.

Regarding acute treatment-related toxicities, such as fatigue, nausea, esophagitis and skin reactions, no patients demonstrated toxicities of Grade 2 or greater. Regarding late toxicities, two patients (4%) experienced Grade 2 radiation pneumonitis (RP) according to the CTCAE, and there were no patients who experienced toxicities of Grade 3 or greater. The V_20_ values of the two patients with RP were 16.9% and 17.5%, respectively, which appeared to be higher than the average of 9.3% (range, 2.2%–19.8%). Six patients had a higher dose area of 75 Gy in the bronchus and the volume ranged from 0.18 ml to 1.52 ml. No patients suffered from treatment-related toxicities regarding the bronchus. No significant differences were observed between patients with a central tumor and those with a peripheral tumor with regard to adverse events, as shown in Table 2.

**Table 2.** Treatment-related toxicities during and after radiotherapy according to CTCAE ver. 4.0

Toxity	Total (*N* = 50)		Central (*N* = 11)	Peripheral (*N* = 39)
	Grade 2	Grade 3 or greater	Grade 2	Grade 2
**Esophagitis**	**2**	**0**	**0**	**2**
**Pneumonitis**	**2**	**0**	**0**	**2**
**Cough**	**4**	**0**	**0**	**4**
**Skin reaction**	**4**	**0**	**0**	**4**
**Rib fracture**	**0**	**0**	**0**	**0**

## DISCUSSION

This prospective study showed the clinical results for patients with surgically inaccessible Stage I NSCLC that was treated by ncIMRT. Excellent 3-year LC and survival rates of 82.6% and 87.1%, respectively, were achieved with minimum treatment-related toxicities. These results were obtained regardless of tumor location, T stage of T1 or T2, age, sex, or whether the tumor was pathologically proven or not, and verified the estimation we made at the starting point. The only significant risk factor was the LC rate between central tumors and peripheral tumors.

In our previous study, we reported that the ncIMRT plan provides high doses of radiation to the margin of the target and reduced doses to the lung compared with non-coplanar planning without intensity modulation for patients with Stage I NSCLC [7]. In IMRT using multi-leaf collimators (MLCs), intensity-modulated beams are made by the summation of many collimated beams, whereas in ncIMRT, intensity-modulated beams are made by compensators. If the target is stable, dose distributions from compensator-based IMRT should theoretically be identical to those of MLC IMRT. However, if the target moves, the former IMRT irradiates parts of the target, and as a result, the target is unevenly irradiated. In contrast, ncIMRT constantly covers the target and therefore the target is evenly irradiated [11–14]. Therefore, ncIMRT is beneficial for the treatment of moving targets. If the power of the linear accelerator increases, ncIMRT may have more benefits over MLC IMRT [15]. Thus, the compensator is well matched with the high-dose-rate flattening filter-free (FFF) beams, and the combination of the compensator and FFF beams enables IMRT to be performed in only a few seconds. Consequently, we believe that patients will be able to undergo ncIMRT under breath-holding conditions, just like when taking a chest X-ray, and also under the inspiration phase, which reduces the irradiated volume of the underlying lung. Therefore, we used the compensator-based system instead of MLC-based IMRT for the moving target. Furthermore, compensator-based IMRT can reduce the number of steps in the quality assurance procedures, because the movement of the MLC does not need to be checked.

Over the last decade, SBRT has become a widely used treatment for patients with inoperable Stage I NSCLC. This therapy concentrates radiation onto small targets using many non-coplanar beams, while only exposing the surrounding tissues to a low dose of radiation. If the low dose of radiation is negligible, the therapy will be ideal. Uematsu *et al.* started hypofractionated radiotherapy for Stage I NSCLC using the SBRT technique, and has obtained highly favorable results [16]. Several studies have shown similar highly favorable LC rates of 83–98%, comparable with those of surgery, with a limited treatment-related toxicity (0–30%) [17–22].

Fractionated radiotherapy is basically used for lung cancer to increase the therapeutic ratios, which are the rates of response against treatment-related toxicities. We believe that fractionated radiotherapy should be more favorable than hypofractionated radiotherapy, even when the SBRT technique is used, because SBRT is not a special therapy but a highly collimated type of radiotherapy. Furthermore, there have been no Phase III studies comparing SBRT and fractionated radiotherapy. Therefore, we treated patients with Stage I NSCLC with usual fractionation using ncIMRT techniques. We determined the ncIMRT dose to be 75 Gy in 30 treatments, covering 95% of the PTV. This schedule of 75 Gy in 30 fractions was calculated to be provided by 93.8 Gy in daily 2 Gy fractions when tumor alpha/beta ratios were assumed to be 10 Gy (equivalent dose). The dose appeared to be almost 100 Gy at the isocenter, and the tumor was irradiated more evenly by the intensity-modulated method than by the usual non-colpanar method [23]. The clinical results were almost equivalent to those of other studies in terms of LC and survival rates. No treatment-related toxicities of Grade 3 or greater were observed for either central or peripheral tumors, although many patients had underlying lung diseases that made it impossible to perform surgery. For Stage I NSCLC, a number of prescription doses achieving ≥100 Gy in equivalent dose showed more favorable LC and survival rates than prescription doses of ≤100 Gy, when the reference point was set at the isocenter [24]. Baardwijk *et al.* evaluated the radiation doses that were required to eradicate Stage I NSCLC, and found correlation between the marginal dose to the PTV and local tumor control by meta-analysis [25]. Concerning central tumors, Timmerman *et al.* reported that patients with central tumors had an 11 times higher incidence of severe treatment-related toxicities than those with peripheral tumors [10]. After his report, several dose fractionations were successfully proposed [26–30]. Senthi *et al.* reviewed 563 central tumors from 20 publications. From the findings, 2- and 3-year LC rates were ≥85% when the prescribed biological equivalent dose was ≥100 Gy. Grade 3 or 4 toxicities developed more commonly following SBRT for a central tumor, but in <9% of patients [31]. In our study, we treated 11 patients with central tumors, but they did not experience any severe adverse events. Investigation of the optimal dose-per-fraction SBRT schedule for patients with central NSCLC is ongoing in RTOG study 0813. Fractionated ncIMRT of 75 Gy in 30 fractions in our study may be one safe alternative option for the treatment of central tumors of Stage I NSCLC, so we used the same dose fractionation for both central and peripheral tumors.

The CALGB 39904 trial prospectively assessed the efficacy of accelerated 3D conformal radiation therapy (3D-CRT) of 70 Gy in 17–29 fractions for patients with early-stage NSCLC. This therapy was well tolerated and severe treatment-related toxicities were not observed in the fraction size of 2.41 Gy or 2.69 Gy [32]. Fang *et al.* reported that the 5-year LC rates for patients with Stage I NSCLC who underwent 3D-CRT with 66 Gy was 70% [33]. Thereafter, dose escalation studies were conducted, but none of them were able to demonstrate superiority due to an increase in adverse events [34–36]. The first randomized Phase II trial comparing SBRT of 66 Gy in 3 fractions with 3D-CRT of 70 Gy in 35 fractions (The SPACE trial) was performed for Stage I medically inoperable NSCLC [37]. No differences between the SBRT and 3D-CRT groups were found in 3-year progression-free survival or OS rates. Our treatment schedule of 75 Gy in 30 fractions was between hypofractionated SBRT of 66 Gy in 3 fractions or 48 Gy in 4 fractions and standard fractionated ncIMRT of 70 Gy in 35 fractions, in the BED calculation [38]. Regarding treatment-related toxicities, incidence rates of esophagitis were significantly higher in the 3D-CRT arm (*P* = 0.006), but this did not apply to pneumonitis or dyspnea. Therefore, the SBRT technique may be effective for reducing treatment-related toxicities.

Many patients had underlying lung diseases, which decreased the threshold of the radiation tolerance dose of their lungs. Recently, low-dose radiation to a wide volume of the lung was pointed out to be a risk factor for RP. Young *et al.* showed that V_5_ was the only significant factor for RP when pulmonary metastases were treated by helical-tomotherapy–based radiotherapy [39]. Low-dose radiation over a large volume may be a risk factor, even if the underlying lung is nearly normal. In our study, 34% of the patients had a history of lung resection, but none of the patients experienced severe lung toxicity (≥ Grade 3). This therapy administers lower doses to the critical organs when the alpha/beta ratio is assumed to be 3 for estimating treatment-related toxicities. This would hence explain the low rates of toxicities.

In our study, 33 tumors out of 50 had no pathological diagnosis, mainly because of underlying lung diseases. These patients were treated for NSCLC, based on the decision of the cancer board. The clinical results were almost comparable with each other by uni- and multivariate analyses. This indicates that the diagnosis is reliable, and this increased reliability should increase the indication of this effective, safe, and less-invasive therapy.

This method continues to come in for occasional criticism as a long-term treatment now that stereotactic body radiotherapy is available in many institutions. However, it supplies effective and safe treatment, even with outdated machines, using compensating filters and no special equipment.

In conclusion, ncIMRT of 75 Gy in 30 fractions to the 95% PTV for patients with Stage I NSCLC is effective and safe, regardless of tumor location.

## References

[1] Raz DJ, Zell JA, Ou SH et al Natural history of stage I non–small cell lung cancer: implications for early detection. Chest 2007;132:193–9. 17505036 10.1378/chest.06-3096

[2] Sawabata N, Miyaoka E, Asamura H et al Japanese lung cancer registry study of 11,663 surgical cases in 2004: demographic and prognosis changes over decade. J Thorac Oncol 2011;6:1229–35. 21610521 10.1097/JTO.0b013e318219aae2

[3] Chang JY, Senan S, Paul MA et al Stereotactic ablative radiotherapy versus lobectomy for operable stage I non-small-cell lung cancer: a pooled analysis of two randomised trials. Lancet Oncol 2015;16:630–7. 25981812 10.1016/S1470-2045(15)70168-3 PMC4489408

[4] Baumann P, Nyman J, Hoyer M et al Outcome in a prospective phase II trial of medically inoperable stage I non-small-cell lung cancer patients treated with stereotactic body radiotherapy. J Clin Oncol 2009;27:3290–6. 19414667 10.1200/JCO.2008.21.5681

[5] Onishi H, Shirato H, Nagata Y et al Stereotactic body radiotherapy (SBRT) for operable stage I non-small-cell lung cancer: can SBRT be comparable to surgery? Int J Radiat Oncol Biol Phys 2011;81:1352–8. 20638194 10.1016/j.ijrobp.2009.07.1751

[6] Louie AV, Palma DA, Dahele M et al Management of early-stage non–small cell lung cancer using stereotactic ablative radiotherapy: controversies, insights, and changing horizons. Radiother Oncol 2015;114:138–47. 25497873 10.1016/j.radonc.2014.11.036

[7] Tajima Y, Nakayama H, Itonaga T et al Dosimetric evaluation of compensator intensity modulation-based stereotactic body radiotherapy for Stage I non-small-cell lung cancer. Br J Radiol 2015;88:20150122. 25996577 10.1259/bjr.20150122 PMC4651373

[8] Cannon DM, Mehta MP, Adkison JB et al Dose-limiting toxicity after hypofractionated dose-escalated radiotherapy in non-small-cell lung cancer. J Clin Oncol 2013;31:4343–8. 24145340 10.1200/JCO.2013.51.5353 PMC3837093

[9] Graham MV, Purdy JA, Emami B et al Clinical dose–volume histogram analysis for pneumonitis after 3D treatment for non–small cell lung cancer (NSCLC). Int J Radiat Oncol Biol Phys 1999;45:323–9. 10487552 10.1016/s0360-3016(99)00183-2

[10] Timmerman R, McGarry R, Yiannoutsos C et al Excessive toxicity when treating central tumors in a phase II study of stereotactic body radiation therapy for medically inoperable early-stage lung cancer. J Clin Oncol 2006;24:4833–9. 17050868 10.1200/JCO.2006.07.5937

[11] Chang SX, Cullip TJ, Deschesne KM et al Compensators: an alternative IMRT delivery technique. J Appl Clin Med Phys 2004;5:15–36. 10.1120/jacmp.v5i3.1965 PMC5723484 15753937

[12] Salz H, Wiezorek T, Scheithauer M et al IMRT with compensators for head-and-neck cancers treatment technique, dosimetric accuracy, and practical experiences. Strahlenther Onkol 2005;181:665–72. 16220406 10.1007/s00066-005-1402-y

[13] Waghorn BJ, Shah AP, Ngwa W et al A computational method for estimating the dosimetric effect of intra-fraction motion on step-and-shoot IMRT and compensator plans. Phys Med Biol 2010;55:4187–202. 20601779 10.1088/0031-9155/55/14/015

[14] O’Daniel JC, Dong L, Kuban DA et al The delivery of IMRT with a single physical modulator for multiple fields: a feasibility study for paranasal sinus cancer. Int J Radiat Oncol Biol Phys 2004;58:876–87. 14967445 10.1016/j.ijrobp.2003.10.031

[15] Ehler ED, Nelms BE, Tome WA On the dose to a moving target while employing different IMRT delivery mechanisms. Radiother Oncol 2007;83:49–56. 17350124 10.1016/j.radonc.2007.02.007

[16] Uematsu M, Shioda A, Tahara K et al Focal, high dose, and fractionated modified stereotactic radiation therapy for lung carcinoma patients: a preliminary experience. Cancer 1998;82:1062–70. 9506350 10.1002/(sici)1097-0142(19980315)82:6<1062::aid-cncr8>3.0.co;2-g

[17] Onishi H, Araki T, Shirato H et al Stereotactic hypofractionated high-dose irradiation for stage I nonsmall cell lung carcinoma: clinical outcomes in 245 subjects in a Japanese multiinstitutional study. Cancer 2004;101:1623–31. 15378503 10.1002/cncr.20539

[18] Nagata Y, Takayama K, Matsuo Y et al Clinical outcomes of a phase I/II study of 48 Gy of stereotactic body radiotherapy in 4 fractions for primary lung cancer using a stereotactic body frame. Int J Radiat Oncol Biol Phys 2005;63:1427–31. 16169670 10.1016/j.ijrobp.2005.05.034

[19] Lagerwaard FJ, Haasbeek CJ, Smit EF et al Outcomes of risk-adapted fractionated stereotactic radiotherapy for stage I non-small-cell lung cancer. Int J Radiat Oncol Biol Phys 2008;70:685–92. 18164849 10.1016/j.ijrobp.2007.10.053

[20] Timmerman R, Paulus R, Galvin J et al Stereotactic body radiation therapy for inoperable early stage lung cancer. JAMA 2010;303:1070–6. 20233825 10.1001/jama.2010.261 PMC2907644

[21] Ricardi U, Filippi AR, Guarneri A et al Stereotactic body radiation therapy for early stage non-small cell lung cancer: results of a prospective trial. Lung Cancer 2010;68:72–7. 19556022 10.1016/j.lungcan.2009.05.007

[22] Fakiris AJ, McGarry RC, Yiannoutsos CT et al Stereotactic body radiation therapy for early-stage non-small-cell lung carcinoma: four-year results of a prospective phase II study. Int J Radiat Oncol Biol Phys 2009;75:677–82. 19251380 10.1016/j.ijrobp.2008.11.042

[23] Fowler JF, Tome WA, Fenwick JD et al A challenge to traditional radiation oncology. Int J Radiat Oncol Biol Phys 2004;60:1241–56. 15519797 10.1016/j.ijrobp.2004.07.691

[24] Onishi H, Shirato H, Nagata Y et al Hypofractionated stereotactic radiotherapy (HypoFXSRT) for stage I non-small cell lung cancer: updated results of 257 patients in a Japanese multi-institutional study. J Thorac Oncol 2007;2:S94–100. 17603311 10.1097/JTO.0b013e318074de34

[25] van Baardwijk A, Tome WA, van Elmpt W et al Is high-dose stereotactic body radiotherapy (SBRT) for stage I non–small cell lung cancer (NSCLC) overkill? A systematic review. Radiother Oncol 2012;105:145–9. 23068707 10.1016/j.radonc.2012.09.008

[26] Stauder MC, Macdonald OK, Olivier KR et al Early pulmonary toxicity following lung stereotactic body radiation therapy delivered in consecutive daily fractions. Radiother Oncol 2011;99:166–71. 21571384 10.1016/j.radonc.2011.04.002

[27] Rowe BP, Boffa DJ, Wilson LD et al Stereotactic body radiotherapy for central lung tumors. J Thorac Oncol 2012;7:1394–9. 22843088 10.1097/JTO.0b013e3182614bf3

[28] Milano MT, Chen Y, Katz AW et al Central thoracic lesions treated with hypofractionated stereotactic body radiotherapy. Radiother Oncol 2009;91:301–6. 19329210 10.1016/j.radonc.2009.03.005

[29] Haasbeek CJ, Lagerwaard FJ, Slotman BJ et al Outcomes of stereotactic ablative radiotherapy for centrally located early-stage lung cancer. J Thorac Oncol 2011;6:2036–43. 21892102 10.1097/JTO.0b013e31822e71d8

[30] Nuyttens JJ, van der Voort van Zyp NC, Praag J et al Outcome of four-dimensional stereotactic radiotherapy for centrally located lung tumors. Radiother Oncol 2012;102:383–7. 22265734 10.1016/j.radonc.2011.12.023

[31] Senthi S, Haasbeek CJ, Slotman BJ et al Outcomes of stereotactic ablative radiotherapy for central lung tumours: a systematic review. Radiother Oncol 2013;106:276–82. 23462705 10.1016/j.radonc.2013.01.004

[32] Bogart JA, Hodgson L, Seagren SL et al Phase I study of accelerated conformal radiotherapy for stage I non-small-cell lung cancer in patients with pulmonary dysfunction: CALGB 39904. J Clin Oncol 2010;28:202–6. 19933904 10.1200/JCO.2009.25.0753 PMC2815709

[33] Fang LC, Komaki R, Allen P et al Comparison of outcomes for patients with medically inoperable Stage I non-small-cell lung cancer treated with two-dimensional vs. three-dimensional radiotherapy. Int J Radiat Oncol Biol Phys 2006;66:108–16. 16904517 10.1016/j.ijrobp.2006.04.015

[34] Sura S, Yorke E, Jackson A et al High-dose radiotherapy for the treatment of inoperable non–small cell lung cancer. Cancer J 2007;13:238–42. 17762758 10.1097/PPO.0b013e31813ffd7b

[35] Bradley J, Graham MV, Winter K et al Toxicity and outcome results of RTOG 9311: a phase I–II dose-escalation study using three-dimensional conformal radiotherapy in patients with inoperable non-small-cell lung carcinoma. Int J Radiat Oncol Biol Phys 2005;61:318–28. 15667949 10.1016/j.ijrobp.2004.06.260

[36] Rosenzweig KE, Fox JL, Yorke E et al Results of a phase I dose-escalation study using three-dimensional conformal radiotherapy in the treatment of inoperable nonsmall cell lung carcinoma. Cancer 2005;103:2118–27. 15830346 10.1002/cncr.21007

[37] Nyman J, Hallqvist A, Lund JA et al SPACE—a randomized study of SBRT vs conventional fractionated radiotherapy in medically inoperable stage I NSCLC. Radiother Oncol 2016;121:1–8. 27600155 10.1016/j.radonc.2016.08.015

[38] Nagata Y, Hiraoka M, Shibata T et al Prospective Trial of Stereotactic Body Radiation Therapy for Both Operable and Inoperable T1N0M0 Non–Small Cell Lung Cancer: Japan Clinical Oncology Group Study JCOG0403. Int J Radiat Oncol Biol Phys 2015;93:989–96. 26581137 10.1016/j.ijrobp.2015.07.2278

[39] Kim Y, Hong SE, Kong M et al Predictive factors for radiation pneumonitis in lung cancer treated with helical tomotherapy. Cancer Res Treat 2013;45:295–302. 24454002 10.4143/crt.2013.45.4.295 PMC3893327

